# Patient participation, a prerequisite for care: A grounded theory study of healthcare professionals’ perceptions of what participation means in a paediatric care context

**DOI:** 10.1002/nop2.106

**Published:** 2017-11-27

**Authors:** Ing‐Marie Carlsson, Jens M. Nygren, Petra Svedberg

**Affiliations:** ^1^ School of Health and Welfare Department of health and nursing Halmstad University Halmstad Sweden

**Keywords:** Children, grounded theory, healthcare professionals, patient participation, paediatric care

## Abstract

**Aims:**

To explore healthcare professionals’ perceptions of what patient participation means in a paediatric care context***.***

**Design:**

A qualitative explorative design with grounded theory.

**Methods:**

Fifteen healthcare professionals who worked in paediatric care settings were either interviewed or asked open‐ended questions in a survey, during December 2015–May 2016. Grounded theory was used as a method.

**Results:**

The study results provide a theoretical conceptualization of what patient participation meant for healthcare professionals in paediatric care and how participation was enabled. The core category “participation a prerequisite for care” emerged as the main finding explaining the concept as ethical, practical and integrated in the care givers way of working. However, the concept was implicit in the organization. Four additional categories illustrated the healthcare professionals’ different strategies used to enhance patient participation; “meeting each child where the child is,” “building a relationship with the child,” “showing respect for each individual child” and “making the most of the moment.”

## 1 INTRODUCTION

Patient participation has been a core concept in the healthcare setting during the last decades and has been considered significant for the patient′s ability to achieve individual goals and satisfaction with care (Dwamena et al., 2012). Moreover, patient participation is also of importance to prevent security risks during care (Weingart et al., 2011). Patient participation is thus, regulated by Swedish national law (Sweden′s Constitution, SFS, 2016:658), which explicitly states that it should be applicable regardless of the context and also cover the whole lifespan.

Studies conducted in healthcare contexts in Western countries have reported that children are a group of patients who are excluded from patient participation, with little attention paid to their views (Runeson, Elander, Hermeren, & Kristensson‐Hallstrom, 2000; Runeson, Hallstrom, Elander, & Hermeren, 2002) and with a marginal role in discussions about their care (Cahill & Papageorgiou, 2007; Coyne, 2006; Moore & Kirk, 2010; Savage & Callery, 2007). Furthermore, research has also found that children are not included when information is given concerning decisions about their care (Coyne, Amory, Kiernan, & Gibson, 2014; Coyne & Gallagher, 2011; Hallstrom & Elander, 2004; Runeson, Martenson, & Enskar, 2007; Runeson et al., 2002) and in terms of their possibilities for being involved in decisions that need to be made about their care (Feenstra et al., 2014; Koller, 2016; Moore & Kirk, 2010; Wyatt et al., 2015). These findings are in contrast to the United Nations Convention on the Rights of the Child (UNCR, 1989) that declares that children and young people have the right to participate in decisions that affect them. Although legislation and conventions on child participation in health care exists, the concept of patient participation, from a child and healthcare professional perspective, has to be understood and implemented in practice (Wyatt et al., 2015). The responsibility for ensuring that these rights are respected in the healthcare context is the duty of healthcare professionals who meet the individual patient (SFS, 2016:658). It is thus, important to investigate the concept of patient participation from the perspectives of paediatric healthcare professionals.

### 1.1 Background

Children want to have a say in issues that affect them and this is true for issues related to their health and health care (Schalkers, Dedding, & Bunders, 2015). As indicated by research findings, children prefer to be a part of their care which means that they actively seeks involvement in consultations with healthcare professionals (Coyne, 2006; Ruhe, Wangmo, Badarau, Elger, & Niggli, 2015). Moreover, when they find themselves in the healthcare context they express a need for information and explanations of what is going on, which enables them to be prepared (Bjork, Nordstrom, & Hallstrom, 2006; Coyne, 2006; Gilljam, Arvidsson, Nygren, & Svedberg, 2016; Runeson et al., 2007; Schalkers et al., 2015; Sjoberg, Amhliden, Nygren, Arvidsson, & Svedberg, 2015). Similarly, children also want to be involved in the decision‐making processes about their care and treatment procedures (Bjork et al., 2006). Several benefits have been found to relate to children′s participation in health care, such as feeling valued and feelings of greater control and less anxiety (Coyne, 2006; Coyne & Gallagher, 2011; Dixon‐Woods, Anwar, Young, & Brooke, 2002). It is clear that there is an increasing awareness of the benefits of child participation, yet it appears that children are rarely or inconsistent involved in healthcare processes (Coyne, 2008; Koller, 2016; Virkki, Heino Tolonen, Koskimaa, & Paavilainen, 2015) and that barriers exists for patient participation in the paediatric healthcare context (Ruhe et al., 2015; Wangmo et al., 2017). Moreover, it has been questioned whether the concept of participation has been fully implemented in healthcare organizations (Coyne & Gallagher, 2011). This may indicate that there are differences between healthcare providers and carers’ views on what patient participation means. To our knowledge, there is a gap in the literature exploring the perspectives of healthcare professionals in paediatric contexts. It is thus, of interest to study what patient participation as a concept means in a paediatric care context.

### 1.2 Aim

The aim of this study was to explore healthcare professional's perceptions of what patient participation means in a paediatric care context.

## 2 METHOD

### 2.1 Design

A qualitative explorative design with grounded theory approach was deemed suitable as it is a method for exploring areas where little is known or when a deeper understanding or new knowledge of an area is desirable (Glaser & Strauss, 1967). Charmaz’ (2014) constructed grounded theory was used in this study since.

### 2.2 Sample and setting

Snowball recruitment was used to acquire a purposive sample of participants. A doctoral student, who worked as a paediatric nurse, took the initial contact and asked healthcare professionals at paediatric clinics for participation in the study. Through interviews with these participants further potential participants were identified and invited to participate in the study. All participants were thus, healthcare professionals currently working with children, 0–18 years old, in a caring context at paediatric clinics in Sweden. Most worked at regional hospitals but five of the participants worked at a university hospital. To ensure a variety of data, several professionals were selected to assure representation of different professionals’ roles at the paediatric clinics; nine paediatric nurses, one generalist nurse two assistant nurses, two social workers and one medical doctor. The participants were all females and had a working experience of paediatric care that varied between 2–30 years.

### 2.3 Data collection

Data were collected through 12 in‐depth interviews performed by the first author and through three surveys with open‐ended questions performed by the second author. All but two of the interviews were carried out face to face; the additional interviews were performed as telephone interviews due to distance. Either digital recording or field notes documented the interviews, based on the participants′ preferences. The interviews lasted between 30–60 minutes and the data collection started with the opening question “Could you please tell me what participation means for you in a paediatric context of care.” Relevant follow‐up probing questions were asked and as data collection and analysis are simultaneous in grounded theory, analytic thoughts and questions that arose from one interview were brought to the next one. Saturation was met when data from a total of 15 participants were collected (Glaser & Strauss, 1967).

### 2.4 Analysis

An ongoing comparison and the simultaneous data collection and data analysis are hallmarks of grounded theory and therefore, an analysis was performed after each interview in accordance with the method′s guidelines (Charmaz, 2014). This analysis started with listening to the interview several times to become familiar with the content. The initial coding continued with analysing data that was coded line by line, though, only the data that fitted the purpose were coded. Where possible, the participants’ own words were used, named in vivo‐coding, which helped the researcher to remain close to the data (Charmaz, 2014). The second step in the analysis was the focused coding, which entails the comparison of codes and data were broken up to components of properties and labelled. These focused codes were labelled in gerunds, as this enables building actions into the data and thus, making processes actions and meanings explicit. The focused codes were subsequently compared with each other and similar codes were sorted and grouped with similar content into categories. The constructed categories were then compared with each other to form tentative categories. After several analyses of the data, a core category that explained the main theme of the data was constructed. In the third step, the theoretical coding, data were collected from new interviews and from the surveys to develop and refine the constructed categories. The interviews continued and data were collected until no new information contributed to the categories, termed “saturation” in grounded theory (Charmaz, 2014). Memo‐writing was used throughout the data collection and analysis in accordance with the method (Glaser & Strauss, 1967). Memos are analytical thoughts and questions asked to the material that may help the researcher to see relationships and shape the analysis and conceptualizing data.

### 2.5 Ethical considerations

The procedures were approved by the regional ethical board (Dnr 2015‐174) and all informants were recruited on a voluntary basis. The confidentiality of interview data and personal identity was assured and the participant′s rights to withdraw from the study at any time was also explained (Declaration of Helsinki, 2013).

## 3 RESULTS

**Figure 1:**
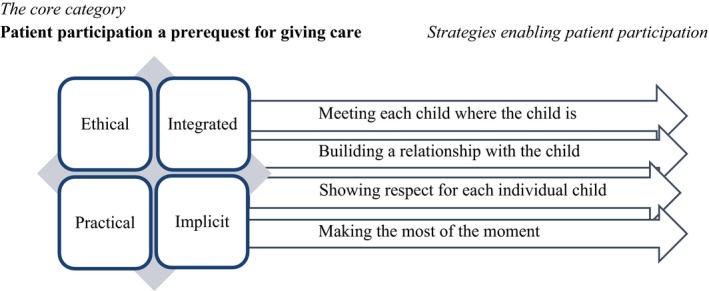
A theoretical conceptualization of what patient participation meant for healthcare professionals in paediatric care and how participation was enabled

The study results provide a theoretical conceptualization that describes what patient participation meant for the healthcare professionals working in a paediatric context. This theoretical conceptualization builds on a core category: “patient participation a prerequisite for care,” which explains how the participants considered patient participation as a necessity to provide ethical and practical care. Moreover, they expressed that patient participation was integrated in the paediatric care, though, the concept seemed to be implicit by the healthcare organization. Furthermore, in addition to this core category, four related categories in the theoretical conceptualization describes strategies of how the healthcare professionals enabled patient participation: (i) “meeting each child where the child is,” (ii) “building a relationship with the child,” (iii) “showing respect to the individual child” and (iv) “making the most of the moment” Figure 1.

### 3.1 The core category “*Patient participation a prerequisite for care*”

The participants expressed that patient participation was a prerequisite for giving care in a paediatric context. This was based on an ethical and practical approach and patient participation was expressed as integrated in the caregiver's way of working. Though, the concept was vague and implicit in the healthcare organization:

The question is not whether the child can participate in their care or not. Instead it is more like, how should I promote participation in the best possible way for this child. (paediatric nurse)

One goal for involving the child was to minimize the distress the care otherwise could cause. If a procedure was to be accomplished, the professionals had to involve and obtain consent from the child. If they would insist on accomplishing something without the child's consent, that would entail a violation of the child's rights:

…if I would do something without the child's approval this would be extremely offensive for them, no matter how old the child is. (paediatric nurse)

It was also considered that the children themselves had to be in charge of the procedure; otherwise, it would not be feasible to carry on throughout the procedure, as children who do not participate react with resistance and protest and become difficult to deal with:

If the child or the parents don′t want to participate, then, there is no way I can go on, nothing will happen and they will leave the room. (paediatric nurse)

However, the participants acknowledged that the degree of children's patient participation varied. They claimed that children were always informed and listened to and were supported in expressing their views. But when procedures and treatments were performed, most of the children were only involved and consulted about relatively trivial matters, such as asking which arm the cannula would be inserted in but not if or when a cannula would be inserted or not, as part of the treatment. Despite patient participation being generally limited, they emphasized that they tried to let the children determine to the extent that they deemed to be possible:

…small children can determine in small matters. (paediatric nurse)

Similarly, the child had sometimes no choice of refusing the procedure or treatment. When moments such as these with lack of freedom of liberty of choice occurred, the healthcare professionals at least tried to provide two alternatives to make the child feel as if they could influence and have an option, even if the healthcare professionals determined these alternatives:

I have to take the blood sample but they may decide how to do this. (paediatric nurse)

The healthcare professionals acknowledged that the child was not generally able to participate to a large extent, e.g. in terms of shared decision‐making about their treatment. When this level of participation did occur, it was more common for older children and children with chronic conditions:

It is possible that you do differently with those who visit us often. Because they know what to expect and they know what to do, so it's possible that you let them participate to a greater extent. (paediatric nurse)

Moreover, the participants discussed the very hierarchical nature of the organization they were working in. There were sometimes different levels of ambition among the team members concerning child participation, which could affect the children's opportunity to be involved. When asked if they used policy documents or knew if the concept was outlined at the organization level, they were vague and most of them gave a negative reply. Moreover, the concept of patient participation was considered as a silent and implicit concept in the healthcare organization. For example, when asked about the meaning of the concept, the healthcare professionals recognized that the word participation was not used or discussed in every day practice, even though they recognized that it was implicit by the healthcare organization that they as professionals should work towards enabling patient participation:

Much of our job is based on documents like the Child Convention and more comprehensive documents of children's right to participate… but I don′t know if there is any principal document at this specific hospital or at this clinic. I don′t know if nurses, or other professions have some documents they follow? However, there is much that is not explicitly expressed, that we are supposed to do and patient participation is one of these. (doctor).

### 3.2 The four additional categories—strategies for enabling participation

Enabling patient participation in a paediatric care context was considered as a complex and time‐consuming issue which required experience and routine acquired from working in paediatric care. The four additional categories demonstrate how the healthcare staff used varying strategies to promote the children's participation.

#### 3.2.1 Meeting each child where the child is

The healthcare professionals emphasized that they initiated participation at each unique meeting with a child. This meant that they had to consider the child′s abilities and capacity, which included the child's age, maturity and health status. To be able to provide the best possible level of participation they adapted their actions to the individual child.

However, most of the participants’ found that the lowest age for child participation was 2–3 years of age:

A one‐year‐old child can decide which arm we should start to examine and what plaster we will put on. But they cannot choose where I shall insert the peripheral venous catheter, that′s up to me to decide. (paediatric nurse)

If they were younger, the parents had a pivotal role functioning as a third part enabling patient participation for their child. This was also true for mentally disabled children where participation was often assigned to the parents. If the parents were unable to be present by their child′s side, it was common that someone among the healthcare professionals acted as a proxy decision‐maker, not as a parent, more like a grown‐up, to protect the child′s right to participate. When asked about how the child′s health status enabled or hindered patient participation, the informants said that even if the child was in palliative care they could participate in some way but this required that the healthcare professionals acted more intuitively, as one informant expressed:

When children are seriously ill, it is difficult and you have to act with a more intuitive feeling to enable participation. (paediatric nurse)

#### 3.2.2 Building a relationship with the child

The category, *‘building a relationshipʼ*, was based on concepts such as trust, interaction and communication. Trust was the foundation of the relationship and had to be earned by the healthcare professionals. The first interaction often started with an eye contact and a smile but play could also be a way to connect with the child. A mutual interaction was needed to enable participation:

You can′t just enter the room with the food tray, you must bring them along with you. (assistant paediatric nurse)

Communication and information were repeatedly mentioned as important for enabling patient participation. At the first meeting with the child, a simple but pivotal question was; “*do you know why you are here today?*” The answer to this question gave a clue as to how much information was needed in this specific situation:

Participation is all about the children understanding why they are at the hospital, otherwise they can′t choose. It must be their question, because you can′t participate if you don't know what it is about. (social worker)

Establishing communication, required skills in adapting oneself to the child′s communication on the latter's own terms. Using an understandable vocabulary or a metaphorical language and to allow communication to take time were considered helpful.

#### 3.2.3 Showing respect for each individual child

The healthcare professionals stated that they acted in the interest of “*nobody else but the child*.” The child was seen as the protagonist and they always turned towards the child when starting to talk. This allowed the child to feel competent and encouraged them to take responsibility for their care:

You have to be super professional and find the right level. If the child has an opinion and the child can express it then you have to deal with it. It's about respect!. (social worker)

Furthermore, patient participation was enabled by showing respect for the child′s preferences:

Children can be very sincere and decide that, now I want to do this…… We try to adopt to each child′s preferences and we really are able to do so since we have a lot of time and less number of. (paediatric nurse)

The interviews provided examples of situations where the children could decide over their body and integrity, by choosing to cover themselves during procedures, or for example, if a girl wore a hijab she could choose whether she would keep it on or not when her weight was taken.

#### 3.2.4 Making the most of the moment

The healthcare professionals maintained that they always supported the child′s patient participation by “*making the most of the moment*” during their care. This meant that they had to be present in their meeting with the child and use the opportunities that occurred in this particular situation:

I can have a picture in my head and suddenly need to change my mind. You know you have to be flexible because it is the child who have to decide. (general nurse)

It required them to inform the child so that he/she understood what would happen but also to include them in the situation. Making the child involved in the situation meant trying to involve the child in everything that happened during their care. When they, for example, were examined they were included in the examination as a co‐examiner; listening to their own heart with the stethoscope, holding the flashlight, pressing buttons or putting the EKG electrodes on to themselves:

First I show the electrodes for them so they can get to know them. Then, I show where I will put them on their body and sometimes I play the buffoon and pretend that I park cars with the electrodes. They can put them on by themselves so that they notice that aren't hurt by them and they can take them off and finally we make a spider together with the electrodes”. (assistant paediatric nurse)

The caregivers stated that there were so few things that the child could influence on so they had to try to enable participation in every possible moment they could even if it just entailed deciding if the child would like to sit or lie down during a procedure.

## 4 DISCUSSION

This article reports on patient participation, a prerequisite for care in a paediatric context. The findings indicate that the concept patient participation is an issue that is both ethical and practical and that is expressed as integrated in the paediatric care. Though, the concept was described by the healthcare professionals as vague and *implicit* in the healthcare organization.

Since the individual healthcare professionals works in an organization that exists in a society and at a certain moment in time, our findings may be interpreted and understood as socially constructed under preexisting structural conditions. Thus, the findings are highlighted in this discussion at three levels: “the discourse of patient participation, patient participation at a structural level and patient participation at the individual level.”

### 4.1 The discourse of patient participation

In this study, children′s patient participation was reported to be well implemented in the paediatric care and viewed as an integral part in the healthcare professionals’ daily way of working; a prerequisite for care. Furthermore, it was acknowledged that most children who wanted to participate were able to do this. The approach adopted here is pleasant and worth aiming for, a discourse linked to the political discourse and to the Convention of the Rights of the Child (Nations, 1989). It is, however, questionable whether this finding is discursively constructed by society and by healthcare professionals. Coad et al. (Coad & Shaw, 2008) have questioned whether children's choice in health care is just rhetoric. Children's involvement in decision‐making processes was only seen in a minority of the narratives in this study and the children did not share power and responsibility for their care which has been proven to be of importance for children (Hallstrom & Elander, 2004). When the participants in our study were asked to provide more explicit examples of how they enabled patient participation, they described that children were always informed and listened to and also supported in expressing their views and similarly involved in procedures. According to Shier′s model of participation (Shier, 2001), it is not sufficient to inform and support children to express their views to be able to call it participation. According to this model, the minimum level of involvement classified as participation is to take children's views into account (Shier, 2001). The healthcare professionals in this study had an ambition to allow as much patient participation as possible but admitted that children were in reality not able to decide very much about their care and when doing so only in small and relatively trivial matters. Our findings are consistent with those of other researchers who describe how children are offered trivial choices but rarely allowed to share power and to take part in decisions that have an impact of their medical care and treatment (L. Moore & Kirk, 2010; Schalkers, Parsons, Bunders, & Dedding, 2016; Virkki et al., 2015). A review of shared decision‐making (SDM) interventions in paediatric care (Wyatt et al., 2015) showed similarly that such interventions did not lead to children's involvement in medical decisions. Although positive examples can be found in our study, there are apparent difficulties in involving children to a higher level of patient participation.

### 4.2 Patient participation at a structural level

To gain a contextual understanding of our results, it is important to declare that the participants worked in an environment characterized by a hospital institution and a hierarchical structure of team members in paediatric care. The healthcare professionals sometimes spoke of patient participation being hindered by varying levels of ambition among the team members and in line with this, functional teamwork has been shown to be important for enabling the decision‐making process (Martenson & Fagerskiold, 2008). If a child‐centred approach is adopted, the care would thus be organized based on the child′s preferences and thereby preventing that differences in ambition can dominate and influence how the care is provided (Coyne, Hallstrom, & Soderback, 2016). The participants in this study describe how individual healthcare professionals can be left alone in their efforts to facilitate patient participation, or receiving minor help and guidance from the organization in their efforts. If there is guidance, it is not evident and has not been explicitly communicated to the staff. We thus, suspect that there are no policy documents at the organizational level that highlight the concept of patient participation. This could reflect the culture of the organization and how they choose to emphasize the importance of patient participation (Longtin et al., 2010). Healthcare organizations need to allocate resources and create policy documents to emphasize and promote children's participation in care (Virkki et al.,^2015^) and paediatric healthcare institutions should adapt guidelines that give practical recommendations about how to understand children's decision‐making capacity (Ruhe et al., 2015). In our study, the individual healthcare professionals tried to “meet each child where the child was,” which included a type of estimation of the child′s capacity, without using any guidelines or tools. Children's levels of participation may thus depend on individual healthcare professionals’ valuations, knowledge and skills. To implement a child‐centred approach successfully in paediatric care, it is thus important to define the knowledge gap in the organization. Furthermore, it is important to take the social systems and characteristics of the organization into account and to develop a clear specification of a clinical practice guideline (Moore et al., 2015; Wensing, 2015). This leads us to suggest that the organization needs to be scrutinized, as it is the organization that has to ensure that the children's rights are fulfilled and that frameworks for how to accomplish this are constructed.

### 4.3 Patient participation at an individual level

It was clear that the individual healthcare professional really tried to facilitate patient participation and used different strategies that enabled participation in everyday practice. One of the categories related to the development of building a trusting relationship, between the child and the healthcare professionals. Similar findings have been reported in earlier studies (Gilljam et al., 2016) who found that children need a trusting and respectful relationship to have the ability to participate in a healthcare situation. To strengthen children's engagement and possibilities to have an impact on the decision process, there is a need not only to listen to them in one‐off events in everyday practice. Instead, there is a need to create a child‐centred arena where the children can express their views, which should be considered by the healthcare professionals (Coyne & Kirwan, 2012).

Another point worthy of note is that the participants highlighted the experience they have had from working in the paediatric context as being helpful in enabling patient participation. There is clearly a possibility that work experience and other factors might have an impact on health professional's ability to facilitate patient participation. It is notable that there appeared to be a variation among the healthcare professionals’ understanding and attitudes about the concept of the different levels of participation. Some were thus contented with enabling trivial matters of participation and gave the impression that this was sufficient. Attitudes among professionals have been shown to be an obstacle for patient participation (Coyne, 2006; Runeson et al., 2002; Virkki et al., 2015). It may thus, be concluded that it is imperative to open up for a conversation about the concept of participation to ensure a more child‐centred approach where power is shifted to the child's advantage in the clinical context.

### 4.4 Strengths and limitations

The *originality* in this study is that we included different professionals that represent the reality children face in a paediatric context. Multiple perspectives validate the phenomena of patient participation and offer new insights of how the discourse of patient participation is constructed from the professional's point of view (Chiovitti & Piran, 2003). However, the small sample size and the homogenous sample with only females were included in the study could constitute a limitation.

*Resonance* in this study was strengthened by discussion of how the findings portray a discourse of patient participation which could be seen as a taken for granted meaning, without reflection, by the professionals (Charmaz, 2014). Finally, *usefulness* is demonstrated in that we go beneath the surface and do not stop with that patient participation is a perquisite for health care. Instead we try to go further by calling for reflection of not just how it should be, but how to enable patient participation in the healthcare environment. The results can thus serve as a valuable reference for healthcare professionals to better understand and develop strategies to enhance children's participation in care.

## 5 CONCLUSION

Healthcare professionals viewed patient participation as an unconditional prerequisite for being able to give care in a paediatric context. The concept was ethical, practical and integrated in the paediatric care. However, they acknowledged that the children mostly participated in small and trivial matters in the care and that the concept was implicit in the organization. There is no research to date, which has explored the discrepancies between the three levels that were discussed here; the discourse, the structured organizational and the individual level. We thus suggest that further research should focus on these levels and on how children's participation in care can be enabled to an extent whereby power is shared.

## 6 RELEVANCE FOR CLINICAL PRACTICE

To ensure qualitative care, patient participation in the paediatric context needs to be raised to a higher level where shared decision‐making is enabled. Therefore, professional caregivers need to have policies from their own organization to realize children's participation in paediatric care. Furthermore, there is still room for improvement at the organizational level to be in the forefront with clinical guidelines for patient participation and to have explicit child rights‐based values that all healthcare professionals could act on. Participatory research including healthcare professionals is needed to highlight the discourse and thereby enable a discussion of how to raise the level of patient participation.

## CONFLICT OF INTERESTS

The authors declared no conflicts of interest with respect to the authorship and/or publication of this article.

## AUTHOR CONTRIBUTION

All the authors have agreed on the final version and meet at least one of the following criteria [recommended by the ICMJE (http://www.icmje.org/recommendations/)]:

- substantial contribution to conception and design, acquisition of data or analysis and interpretation of data;
- drafting the article or revising it critically for important intellectual content.
